# Spiroplasma species as a rare cause of congenital cataract and uveitis: a case series

**DOI:** 10.1186/s12886-021-02201-0

**Published:** 2021-12-15

**Authors:** Navid Farassat, Michael Reich, Annerose Serr, Sebastian Küchlin, Marwa Erwemi, Claudia Auw-Hädrich, Hermann Krastel, Wolf Alexander Lagrèze

**Affiliations:** 1 Eye Center, Medical Center, Faculty of Medicine, Albert-Ludwig University Freiburg, Killianstraße 5, 79106 Freiburg, Germany; 2 Institute for Microbiology and Hygiene, Faculty of Medicine, University of Freiburg, Freiburg, Germany; 3 Department of Ophthalmology, Medical Faculty Mannheim, Ruprecht-Karls-University Heidelberg, Heidelberg, Germany

**Keywords:** *Spiroplasma ixodetis*, Congenital cataract, Uveitis

## Abstract

**Background:**

To date, only four cases of ocular spiroplasma infection have been reported in the entire ophthalmic literature. We add two more cases to raise awareness of this sight-threatening congenital disease that manifests as cataract with ocular inflammation.

**Case presentation:**

Both infants were referred for cataracts associated with ocular inflammation. Case 1, a 3-week-old neonate presented with unilateral cataract, ocular inflammation and elevated intraocular pressure. Case 2 was a 3-month-old infant with bilateral cataract and panuveitis. Lensectomies with or without vitrectomy and subsequent analyses of the specimens were performed. Transmission electron microscopy and multiplex polymerase chain reaction or 16 s rRNA gene polymerase chain reaction revealed spiroplasma species.

**Conclusions:**

Spiroplasma as a very rare cause for congenital cataract might be underdiagnosed. We recommend performing polymerase chain reaction to probe for spiroplasma species in congenital cataracts with an inflammatory component.

**Supplementary Information:**

The online version contains supplementary material available at 10.1186/s12886-021-02201-0.

## Background

Congenital cataract is a leading cause of childhood blindness [1]. While most cases occur on a genetic or maldevelopmental basis, the exact etiology may remain elusive in the context of ocular inflammation. Infectious causes of congenital cataracts are often summarized with the acronym TORCH. Common pathogens of the TORCH group are toxoplasmosis, rubella, CMV and herpes simplex. The “O” represents other rare pathogens. We here report two cases of congenital cataract and uveitis due to spiroplasma species to raise awareness for this rare sight-threatening condition. One of these cases represents the first in the literature to show not only inflammation of the anterior segment but also of the posterior segment of the eye.

## Case presentation

### Case 1

**Fig. 1:**
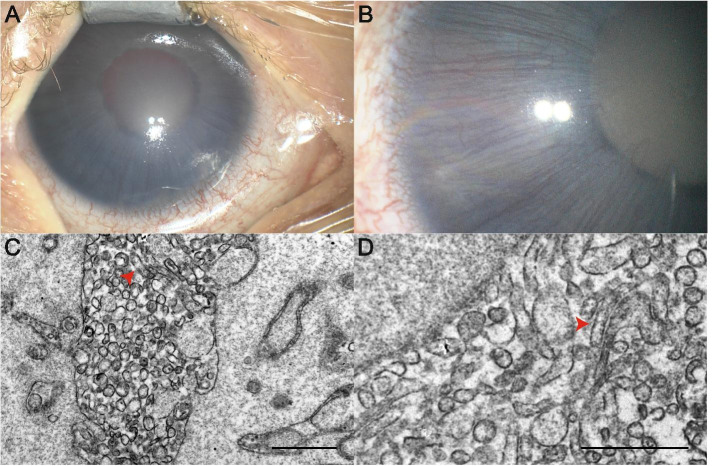
Case 1. **A**, **B** Anterior segment OD of patient 1 showing a diffusely opacified lens and a hyperemic iris with straight and prominent vessels extending onto the lens. **C**, **D** Transmission electron microscopy images of crystalline lens material revealing multiple intracellular, rod-shaped, filamentous and round-shaped microorganisms (red arrowheads), scale bar = 1 μm

A slightly premature born (35w 5d), 3-week-old, otherwise healthy infant was referred for a white fleck OD after inconspicuous pregnancy. Family history for congenital cataracts was negative. Examination revealed anterior uveitis with a very shallow anterior chamber, circular anterior synechiae in the chamber angle, a delicate fibrine pupillary membrane with posterior synechiae and a diffuse whitish opacification of the entire lens. The iris was hyperemic with straight and prominent vessels extending slightly onto the lens (Fig. 1A, B). Initial intraocular pressure (IOP) measured 26 mmHg OD. Corneal diameter OD was increased to 11.5 mm (10 mm OS) and axial length was 19.2 mm OD (17.1 mm OS). After a 5-week course of a topical and systemic anti-inflammatory, antibiotic and IOP-lowering therapeutic regimen, synechiolysis and lensectomy were performed. The vitreous was not infiltrated and the retina appeared normal. Dexamethasone, vancomycin and ceftazidime (each 0.1 ml of vancomycin 10 mg/ml, ceftazidime 20 mg/ml, dexamethasone 4.44 mg/ml) were injected intravitreally. Postoperatively, the patient received tapering topical steroids. Polymerase chain reaction (PCR; comprising panfungal PCR, Toxoplasma gondii qPCR, eubacterial PCR, Herpes simplex 1/2 PCR, Chlamydia trachomatis qPCR, Myoplasma genitalium qPCR, Mycoplasma hominis qPCR, Ureaplasma urealyticum/parvum qPCR and Mycoplasma multiplex n-PCR) of the lensectomy probes was performed. Mycoplasma multiplex n-PCR revealed spiroplasma species. 16 s rRNA gene PCR and sequencing identified spiroplasma ixodetis as the subspecies. Other infectious causes of congenital cataracts were ruled out either through PCR of lens specimens (Herpes simplex, Toxoplasmosis), serologically (CMV) or clinically (Rubella). Transmission electron microscopy (TEM) of lens fragments collected without centrifugation visualized intracytoplasmic irregular, filamentous and round microorganisms matching previous reports of spiroplasma (Fig. 1C, D). Postoperatively, a contact lens was fitted and occlusion therapy was initiated.

### Case 2

**Fig. 2:**
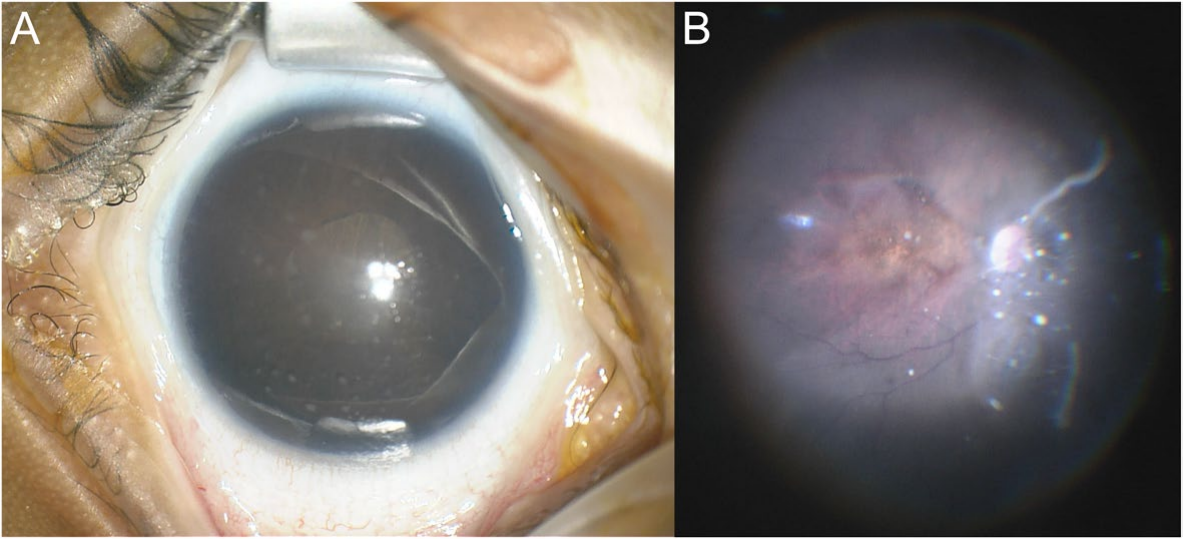
Case 2. **A** Anterior segment OD of patient 2 showing a diffuse cataract and corneal precipitates. **B** Intraoperative funduscopic image OD revealing vitreous infiltration

At the age of 3 months, a full-term male infant was referred for bilateral cataracts OU. Family history for congenital cataracts was negative. We found diffuse white cataracts obscuring the fundus OU. Concomitant inflammation of the anterior segments with corneal precipitates, posterior synechiae and small pupillary granulomata OU was detected (Fig. 2A). Ultrasonography showed infiltration of the vitreous and attached retinas OU. IOPs were normal. Lensectomy and synechiolysis was performed sequentially OU. Intraoperatively, the vitreous showed mild cellular infiltration and snowballs. The peripheral retina exhibited few whitish, partially necrotic lesions (Fig. 2B). Postoperatively, systemic therapy was initiated with intravenous methylprednisolone 30 mg/kg/d for 3 days and oral erythromycin for 21 days. Topical steroids were tapered within 3 weeks. PCR (comprising panfungal PCR, Toxoplasma gondii qPCR, Bartonella henselae n-PCR, Mycobacterium n-PCR, M. tuberculosis complex n-PCR, eubacterial PCR, and Mycoplasma multiplex n-PCR) of vitreous probes revealed spiroplasma species. Subspecies identification through 16 s rRNA gene PCR was not performed. Other infectious causes of congenital cataracts were ruled out through PCR of vitreous probes (Toxoplasmosis, CMV, Rubella). TEM of a vitrectomy centrifugate showed rod-shaped structures matching spiroplasma. However, this mode of specimen preparation did not allow for high quality imaging and therefore these images are not presented herein. Five years after lensectomies, visual acuity was 20/200 OD and 20/100 OS with aphakia spectacles for optical correction. Aphakic glaucoma did not occur.

## Discussion and conclusions

We present two cases of congenital cataract and concomitant intraocular inflammation due to infection with spiroplasma species. Spiroplasma is a small helical genus of mollicutes, a type of intracellularly growing bacteria without a cell wall. It colonizes plants, arachnids and insects and has been shown to exhibit a mostly symbiotic, rarely pathogenic interaction with its hosts [2]. Little is known about the transmission between animal and human hosts. Nevertheless, spiroplasma ixodetis has been related to four cases of congenital cataract with anterior uveitis, suggesting intrauterine transmission [3, 4]. Two patients each were affected either uni- or bilaterally. Lorenz et al. were the first to report such a case in 2002 [3]. In contrast to these reports, patient 2 in this study showed inflammation not only of the anterior but also of the posterior segment. This is in accordance with experimental studies: Spiroplasma mirum, isolated from rabbit ticks in Georgia, USA, also known as the suckling mouse cataract agent (SMCA), caused panophthalmitis and microphthalmia in newborn mice, rats, hamsters and rabbits when inoculated intracerebrally. Of note, adult animals were resistant [5–7]. Likewise, there are no reports of ocular spiroplasma infections in adult humans. However, three reported cases of systemic infections in immunocompromised adult patients indicate that spiroplasma species do not exclusively affect infants [8, 9, 10].

Spiroplasma as a very rare infectious cause of congenital cataract might be underdiagnosed, as neither specific serology, PCR nor TEM are part of routine diagnostics. We therefore recommend performing PCR to probe for this entity in congenital cataracts with an inflammatory component.

## Supplementary Information

**Additional file 1.**

## Data Availability

The datasets used and/or analysed during the current study are available from the corresponding author on reasonable request.

## Abbreviations

IOP: Intraocular pressure
PCR: Polymerase chain reaction
SMCA: Suckling mouse cataract agent
TEM: Transmission electron microscopy
