# Supplementary figures and images for: Spiroplasma species as a rare cause of congenital cataract and uveitis: a case series
Source: BMC Ophthalmol. 2021 Dec 15;21:434. doi: 10.1186/s12886-021-02201-0 (PMC8672502; doi:10.1186/s12886-021-02201-0)

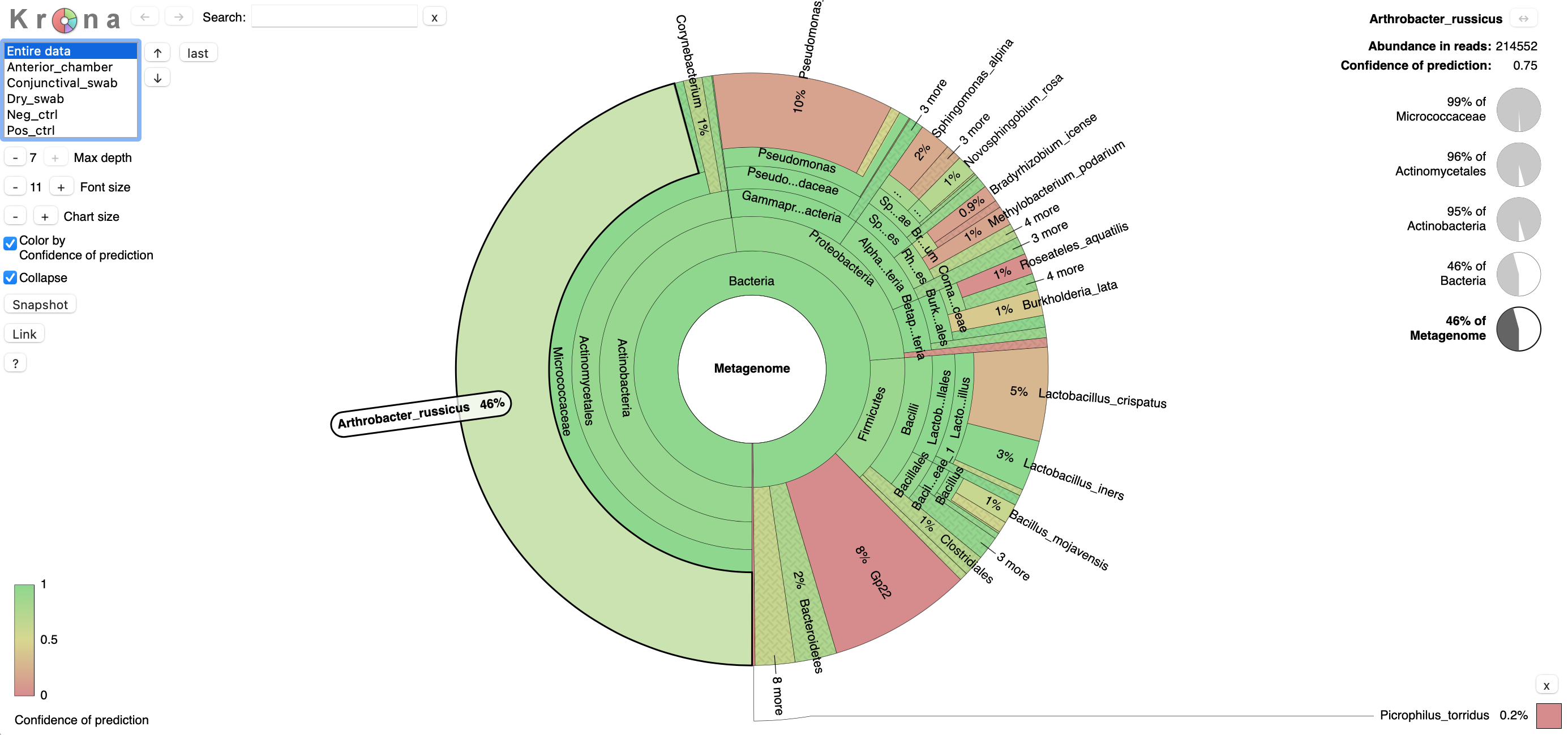

Supplement: Supplementary file 1 — Additional file 1. [file 12886_2021_2201_MOESM1_ESM.png]
